# Discontinuation of Cerebro-Spinal Fluid (CSF) Drainage in Acute Hydrocephalus: A Prospective Cohort Study and Exploratory Data Analysis

**DOI:** 10.3390/neurosci5040030

**Published:** 2024-10-08

**Authors:** Anand S. Pandit, Joanna Palasz, Lauren Harris, Parashkev Nachev, Ahmed K. Toma

**Affiliations:** 1 Victor Horsley Department of Neurosurgery, National Hospital for Neurology and Neurosurgery, London WC1N 3BG, UK; 2 High-Dimensional Neurology, Queen Square Institute of Neurology, University College London, London WC1N 3BG, UK; 3 Department of Neurosurgery, Queen’s Hospital, Rom Valley Way, Romford RM7 0AG, UK

**Keywords:** acute hydrocephalus, drain, weaning, CSF, subarachnoid haemorrhage, neurocritical care

## Abstract

Background: The optimal management of CSF drainage in acute hydrocephalus, in particular when to initiate drain weaning, remains uncertain. This study aimed to evaluate the impact of timing and method of drain weaning on patient outcomes. Methods: This prospective observational study in a large-volume tertiary neuroscience centre included all adult patients who required temporary CSF drainage for acute hydrocephalus of any cause between January 2020 and March 2021. Contemporaneous data collection was conducted, including patient demographics, time to clamp, weaning methods, and clinical outcomes of hospital length of stay (LOS), rate of shunt insertion, drain-related infections, and mechanical complications. Univariate and multivariate statistical analyses were performed to identify the independent associations of timing-related factors. Results: A total of 69 patients were included (mean age = 59.4 years). A total of 59% had CSF diversion for aneurysmal subarachnoid haemorrhage, and 88% had EVD drainage. The length of drainage prior to the first clamp was significantly associated with the overall length of drainage (*p* < 0.0001), LOS (*p* = 0.004), and time to shunt (*p* = 0.02) following multivariate adjustment. For each day delayed in initiating the drain challenge, the overall LOS increased by an additional 1.25 days. There was no association between the weaning method and LOS, the rate of shunting, or CNS infection; however, those in the gradually weaned group had more mechanical complications, such as drain blockage or CSF leakage, than those rapidly weaned (*p* = 0.03) after adjustment. Discussion: This study recommends challenging the drain early via a rapid wean to reduce LOS, mechanical complications, and possibly infections. The consequences of temporary CSF diversion have significant implications at financial and patient levels, but the quality of evidence regarding weaning remains poor. Further randomised multicentre studies and national databases of practice are required to allow definitive conclusions to be drawn.

## 1. Introduction

Temporary cerebral spinal fluid (CSF) diversion with an external ventricular drain (EVD) or a lumbar drain (LD) are common neurosurgical procedures used in the treatment of acute hydrocephalus. This is often secondary to subarachnoid haemorrhage (SAH), intracerebral haemorrhage (ICH), intraventricular haemorrhage (IVH), traumatic brain injury (TBI), meningitis, shunt failure, or ventricular obstruction, e.g., by tumours [1,2]. CSF drainage is associated with complications, notably infections, malposition, and obstruction, leading to morbidity, mortality, and increased healthcare costs [3,4].

Optimal management of CSF drainage, in particular when to initiate drain weaning and the rate of weaning, is uncertain, with a lack of high-quality evidence guiding recommendations. Weaning strategies can be rapid or gradual, with no standardised definitions. It is unclear whether a rapid or gradual wean impacts the rate of infections, mechanical complications, or permanent CSF diversion with a ventricular shunt.

Most of the literature focusses on EVDs after SAH. A survey in North America and Europe exposed the huge variability in weaning methods within institutions and between clinicians [5,6]. Weaning is currently based on the experience of the treating consultant or individual patient’s criteria rather than standardised cessation protocols [6]. At present, gradual drain weaning is the most common strategy. However, the American Stroke Association (ASA) and Neurocritical Care Society recommend rapid weaning, extrapolating the results from one randomised controlled trial (RCT) [7,8,9]. The literature regarding weaning strategies for ICH, IVH, or TBI is scarce, with limited evidence favouring one method over the other [10,11,12,13].

This prospective cohort study evaluated the timing and method (rapid or gradual) of drain weaning in patients with acute hydrocephalus from any aetiology in a large-volume tertiary neurosciences centre. It assessed whether the weaning method impacted overall hospital length of stay, rate of shunt insertion, drain-related infections, and mechanical complications.

## 2. Methods

### 2.1. Reporting Guidelines

This article adheres to Strengthening the Reporting of Observational Studies in Epidemiology (STROBE) guidelines for reporting cohort studies [14].

### 2.2. Ethics

The local institutional review board approved this study as an ongoing service evaluation of neurosurgical patients with acute hydrocephalus at our centre. Informed consent for drain procedures was obtained for all patients.

### 2.3. Participants

This study was conducted in a large-volume tertiary neurosciences centre in London, United Kingdom. Included were all adult patients (>18 years old) who were admitted between January 2020 and March 2021 and who required placement of a ventricular or lumbar drain to treat acute hydrocephalus. The decision to proceed with drain insertion was based on clinical and radiological evidence of acute hydrocephalus confirmed by the consultant neurosurgeon on-call. The decision on which choice of drain device (tunnelled-EVD, bolt-EVD, or LD) was made by the neurosurgical consultant caring for each patient and was not specified by a study protocol. All medical decisions were made in accordance with the local guidelines and standard operating procedures. Patients were excluded if they had a proven extra-cranial infection prior to drain insertion (febrile or elevated inflammatory markers with a confirmed source, e.g., urine or chest), had chronic hydrocephalus, required a drain for treatment of CSF leakage secondary to operative complications, had prophylactic drain placement (e.g., as a short-term safety mechanism following an endoscopic third ventriculostomy or posterior fossa tumour resection), had more than one type of drain inserted (for example, an EVD followed by a lumbar drain), or died during their admission and CSF drainage (in an attempt to limit confounders).

### 2.4. Data Collection

Patients with temporary drains in situ were prospectively assessed on a daily basis and documented ensuring a high degree of accuracy unlikely to be achievable with retrospective assessment. Patient-level data were retrieved from the institution’s electronic health record (Epic System Corporation, Madison, WI, USA) and the electronic patient referral system (referapatient.org). These data encompassed clinical documentation from medical, nursing, and auxiliary teams, laboratory investigations, radiological reports, and operation notes. Drain parameters, including choice and timing of device, volume and pressure settings, placement issues, and the incidence of mechanical complications, were also recorded and verified in person by a member of the study team (J.P.). Other variables’ data were extracted contemporaneously for every patient on the day of or the morning after admission. All data was quality-checked and vetted by a separate member of the study team (A.S.P.). Other members of the neurosurgical team were unaware of this daily data collection to avoid any Hawthorne effect [15].

Drains were placed by the resident neurosurgeon either in the operating theatre or at the bedside in the neurosurgical intensive care unit (ITU). Tunnelled EVDs (ARES, Medtronic, Minneapolis, MN, USA) were inserted in the operating theatre under general anaesthesia, while bolted EVDs (Spiegelberg Silverline, Hamburg, Germany) were inserted in either the operating theatre or ITU. Both types of EVD were directed with the tip toward the ipsilateral lateral ventricle frontal horn. Lumbar drains (Spiegelberg Silverline, Hamburg, Germany) were inserted in theatre or at the ITU bedside with or without sedation, and the stylet was inserted at the L3/L4 or L4/L5 intervertebral space.

### 2.5. Weaning

**Figure 1:**
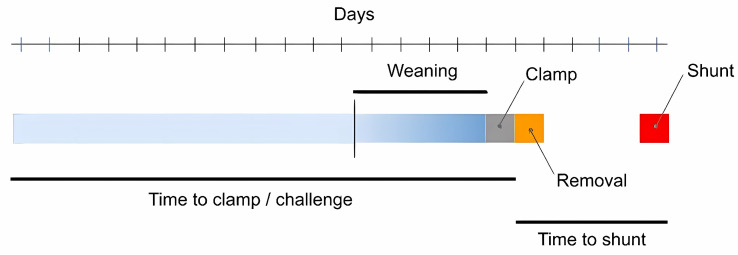
Example timeline of a patient undergoing a gradual wean followed by shunt insertion.

Patients who had a rapid wean were clamped immediately, regardless of the preceding drain pressure. These patients would then be monitored for 24 h, and a CT head scan would be obtained. If the patient demonstrated either clinical and/or radiological signs of hydrocephalus, then the challenge would have failed, and the drain would be unclamped. Patients who had a gradual wean were weaned in any format other than the above, most commonly with an interval increase in wean pressures in the few days prior to the drain being clamped (Figure 1). Differences in weaning were based on attending preference in the absence of clear evidence-based guidelines rather than being selective between patients (Supplementary Methods).

Some patients had their drains removed either unintentionally by themselves or accidentally during patient transfers or turning. These patients thus ‘challenged’ themselves and, as for the above groups, were assessed clinically and radiologically in the following 24 h.

### 2.6. Outcomes

Our aim was to compare the effects of both the weaning method and early versus later clamping on patient outcomes. Our primary outcome was the total hospital length of stay (LOS), and secondary outcomes included timing and frequency of shunt placement and incidence of drain-related complications. Drain-related complications included an infection or mechanical drain issues. A central nervous system infection was confirmed if the patient had a positive CSF microbiological culture or was started on CNS-targeted antibiotic therapy—both under the purview of a senior attending microbiologist with a specialism in drain-associated infections. Mechanical complications included drain blockage, dislodgement, and CSF leak bypassing the drain.

### 2.7. Data Analysis

To determine the appropriate univariate statistical test, normality testing was first performed by inspection of the relevant data histogram and Kolmogorov–Smirnov test.

Statistical modelling was performed to determine the relationship between predictor variables and the outcome in two stages. First, univariate testing was performed using appropriate parametric or non-parametric methods. Second, multivariate approaches were applied to estimate adjusted effects using the following steps for both linear and logistic methods. Predictor variables included patient diagnosis, age, sex, time to first clamp, wean method, drain type, number of drains, and type of drain collecting system. Given the large number of possible predictors and confounders that may explain an outcome of interest, a permutation approach was used to find the model with the lowest Akaike and Bayesian Information Criterion (AIC/BIC). Where these did not discriminate between models, adjusted R^2^ was used to determine the most parsimonious model with the best fit. Regression method assumptions such as equal variance or homodescascity were tested using appropriate tests and by inspection of the residual-prediction plot.

Non-adjusted and adjusted coefficients and *p*-values are presented along with correlation plots to demonstrate the relationship between the predictor variables of key interest (wean method and time to first drain challenge/clamp) and the outcome after controlling for the effect of other independent variables. Where several post hoc tests were performed, the Holm–Bonferroni adjustment was used to correct for multiple comparisons. 

## 3. Results

### 3.1. Demographics

A total of 69 patients met our inclusion criteria for entry into this study, with a total of 80 drains inserted (Table 1). The diagnosis for the majority of patients was acute hydrocephalus secondary to aneurysmal or non-aneurysmal SAH (62.3%), although there were non-trivial numbers of obstructive hydrocephalus secondary to stroke (21.7%) or tumours (13.0%). Of the patients with obstructive hydrocephalus, the majority (>75%) had tri-ventriculomegaly, while the remainder had isolated ventriculomegaly of either the lateral or third ventricles. Three patients were excluded from certain outcome analyses as they had prolonged lengths of stay (greater than two standard deviations from the mean of the cohort). This was due to complex discharge and rehabilitation reasons.

Eight patients had serial drainage (i.e., one drain was removed and another of identical type was inserted consecutively), while three patients had concurrent bilateral EVDs.

### 3.2. Wean Patterns

**Figure 2:**
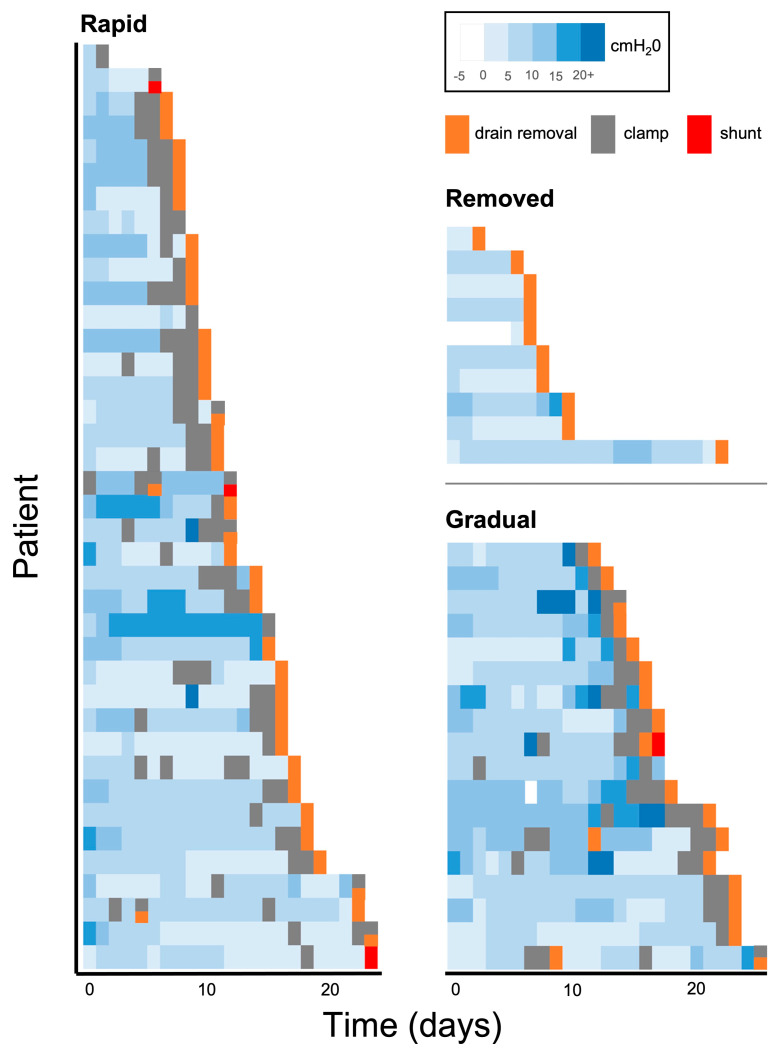
Individual patient Gantt intensive care unit timeline from drain insertion to drain removal is categorised by the weaning method. Note patients may be shunted after discharge from the ICU and beyond this timeline. Legend (top right) demonstrates days with different levels of set pressure (blue) and whether the drain was removed (orange), clamped (grey), or shunted (red).

The differences between individual patients in terms of time to wean and timing of clamp are illustrated in the Gantt chart (Figure 2). A summary of unadjusted and adjusted key results is given in Table 2.

### 3.3. Influence of Weaning on Hospital Length of Stay

**Figure 3:**
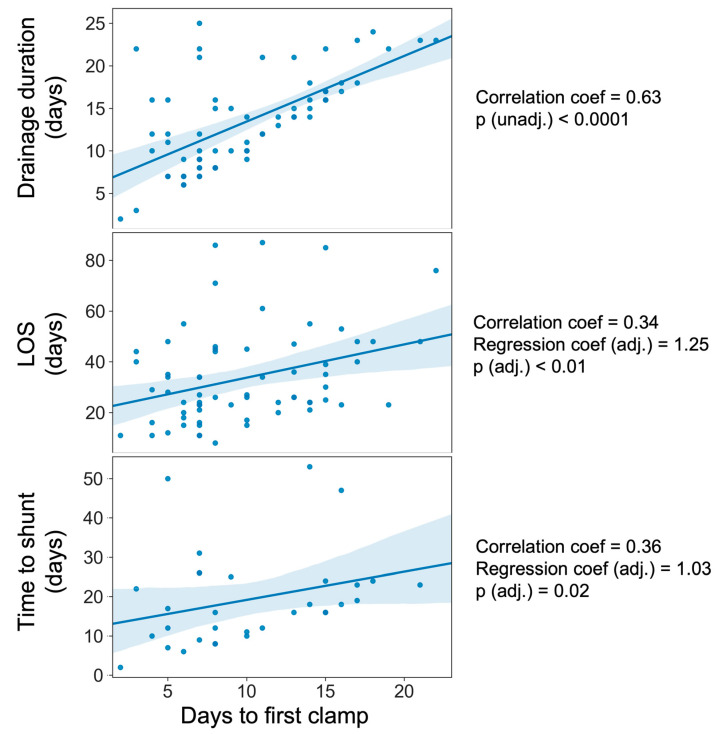
Scatter plots demonstrating days to first clamp or removal against primary and secondary outcomes. (LOS = length of stay; blue line = line of best fit with confidence intervals shown in light blue).

On univariate testing, no significant differences were found between the weaning method and hospital LOS (Supplementary Table S1). As expected, the length of drainage prior to the first clamp was significantly associated with both overall length of drainage (Spearman’s Rank coefficient = 0.63, *p* < 0.0001) and hospital LOS (Spearman correlation = 0.34, *p* = 0.004) [Figure 3]. After adjustment for confounders, including the occurrence of complications, this latter association remained significant (coefficient = 1.25, t = 2.73, *p* = 0.008) [Table 2 and Supplementary Table S2]. In other words, after holding other variables constant, for each day delayed in clamping the drain or initiating the drain challenge, the overall length of stay increased by an additional 1.25 days (or an additional 25% beyond the day lost due to drainage).

### 3.4. Influence of Weaning on Shunt Frequency and Timing

The weaning method (rapid vs. gradual) was not significantly associated with the occurrence of shunting on uni- or multivariate analysis. Of those patients who were shunted, there were significant differences between groups in the time taken to shunt (Supplementary Table S3), with patients gradually weaned waiting the longest time to be shunted (median = 23.5 days). Following post hoc Dunn testing, significant differences were found between ‘gradual’ weaning and ‘removed’ subgroups, with the latter having the shortest time to shunt (median = 10 days). This relationship approached significance after adjustment (Supplementary Table S4) and can be further visualised using Kaplan–Meier survival curves (Supplementary Figure S1), although these were not found to be significantly different.

Time to first clamp was not associated with the occurrence of shunting but was positively correlated with the time taken to shunt (Spearman’s Rank coefficient = 0.36, *p* = 0.04) [Figure 3]. After multivariate adjustment (Table 2, Supplementary Table S4), this remained significant (coefficient = 1.03, t = 2.46, *p* = 0.02).

### 3.5. Influence of Weaning on CNS Infection

The weaning method was not associated with the occurrence of CNS infection (or treatment for it), but of those who were considered to have an infection, there were significant differences between groups when that infection occurred (KW, statistic = 11.15, *p* = 0.003) [Supplementary Table S5]. Post hoc differences were found between gradually weaned patients, whose median time to infection was 11 days, compared to those rapidly weaned (median = 5.5 days, Dunn’s test, *p* = 0.008). This relationship persisted after multivariate adjustment (rapid weaning, coefficient = −6.31, t = −5.0, *p* < 0.0001) [Table 2 and Supplementary Table S6]. Days to the first clamp were not linked with the occurrence of CNS infection nor time to infection either on uni- or multivariate analyses [Supplementary Table S6].

### 3.6. Influence of Weaning on Mechanical Drain Complications

Given that by default, most patients in the ‘removed’ wean group had a dislodgement or accidental retraction, i.e., a mechanical complication—these patients were excluded from this sub-analysis. There were significantly more discrete mechanical complication episodes in gradually weaned versus rapidly weaned patients (MWU, statistic = 427, *p* = 0.04) [Supplementary Table S7], which persisted in significance after multivariate adjustment (rapid weaning, coefficient = −0.54, t = −2.19, *p* = 0.03) [Table 2 and Supplementary Table S8]. However, the time taken to the first mechanical failure did not significantly vary by the weaning method. Time to first clamp was neither associated with time to first mechanical failure, the occurrence of any mechanical failure, nor the number of mechanical episodes on uni- or multivariate analyses.

## 4. Discussion

### 4.1. Summary of Results

The optimal weaning strategy for temporary CSF drainage in acute hydrocephalus is uncertain, with large variations at an institutional and physician level, at least in part driven by a lack of high-quality evidence [4]. This study found that the length of hospital stay increased with a delay in initiating the drain challenge, surplus to the days that the drain was in. Rapid weaning reduced mechanical complications, although the weaning strategy did not impact hospital length of stay or rate of shunt insertion. While there was no difference in overall drain-related infections, those who were gradually weaned had infections later.

### 4.2. Principal Findings and Interpretation

In our study, for each day delayed in initiating the drain challenge, the overall length of stay increased by an additional 1.25 days. This is consistent with other literature, where the longer it takes to perform the first wean trial, the longer the patient will remain in the ITU and in the hospital [16].

In our study, the weaning method itself was not associated with the length of stay before or after multivariate adjustment. There is one prospective RCT comparing rapid and gradual weaning of EVDs in 81 patients with SAH [9]. Similar to our study, in this trial, the rapid wean was completed in 24 h, with EVD clamping immediately on initiating weaning, with a confirmation CT scan at 24 h if successful. In contrast, the gradual wean lasted 4 days, starting at 15 cm H_2_O for 24 h, raised by 5 cm each morning, and clamped on day four. This found an increased length of stay in the gradually weaned group by univariate comparison. This is likely due to the number of days the weaning adds to the total length of drainage in the RCT, whereas in our study, we looked independently at the weaning method and the time to clamp using multivariate analyses. Other multicentre studies have been inconclusive, with some supporting a significantly increased hospital length of stay in the gradually weaned group [17] and others showing no difference [16].

A total of 46% of patients in our study were shunted. The weaning method was not associated with the frequency of shunting, although those who had their drains removed accidentally were associated with shunting at earlier time points. The literature on the effect of the weaning strategy on shunt rates is also uncertain. The aforementioned RCT found no difference between the rapidly weaned and gradually weaned groups, although shunt rates were very high across both (63.4% and 62.5%, respectively). Here, a shunt was placed in all patients on first weaning failure, in contrast to this study, which permitted multiple drain challenges. A 2022 prospective study of three centres with 139 patients found the rate of shunting following EVD insertion for SAH was 32% for the rapid wean group versus 39% for the gradual [16]. Limiting direct comparisons with our results was that definitions of rapid and gradual weaning were inconsistent both in study design and between their own participating centres. Two of the three centres defined a rapid wean as immediate closure of the drain (like our study), and one defined as closure of the drain within 48 h, with a non-trivial proportion (21%) crossing over from gradual to rapid groups and 4% vice versa. Results from retrospective studies were also inconclusive. Rao et al. demonstrate, using multivariate logistic regression, that a rapid wean is associated with fewer shunts [18]. In contrast, Jabbarli et al. found a significant independent association between a rapidly weaning regime and greater shunt dependency.

If the influence of the weaning method on shunt frequency remains equivocal, in support of a rapid wean is the significantly reduced frequency of mechanical complication episodes in this subgroup, although this may be collinearly related to the length of drainage. Similar to this cohort, mechanical complications of CSF diversion, including blockage, leakage, and dislodgement, have been found to occur in approximately 39% of cases [19]. Additionally, a rapid wean may lead to earlier mobilisation, decreasing complications of prolonged bed rest, including pressure ulcers, aspiration pneumonia, and muscle atrophy [20,21]. Drain output and clamp failures were also significant predictors of shunt placement [22], although these factors were not assessed specifically in our analyses.

The risk of drain-related infections has been related to the duration of catheterisation [23,24]. These can potentially lead to ventriculitis, brain abscesses, and negative patient outcomes, including death. In the UK, the EVD-related infection rate was approximately 9.3%, with a higher risk if drainage was more than 8 days [25]. In Canada, the rate was 9.5%, occurring at a mean of 8.9 days [26]. In our study, there was no difference in the incidence of infection between weaning groups, although this may be a reflection of a more sensitive but less specific classification of CNS infection with higher rates of infection in our cohort (29%) and/or insufficient power of our study to detect this. Patients who were gradually weaned had infections at later time points than those who were rapidly weaned, independent of the length of drainage prior to clamping.

### 4.3. Limitations and Strengths

This is a prospective study with detailed, contemporaneous data collection, allowing precise, accurate results. The definitions of weaning were clearly defined, and components were examined separately—the initiation of weaning, the method of weaning, and the overall length of drainage. The method of weaning is non-standardised and variable in the literature, which makes comparisons difficult. Several limitations can be identified. First is the monocentric study design of 69 patients. This study had a 14-month patient inclusion period only due to the resource demands for detailed, daily data collection. Comparison groups were relatively small, with only 19 patients in the gradually weaned groups. Despite this, results were significant, suggesting the strength of association would only increase in a larger sample size.

This was a pragmatic study with a heterogeneous patient group, including those with different aetiologies underlying the acute hydrocephalus and different drainage methods, including both EVD and lumbar drains. Like the literature, the majority (59%) of patients presented with acute hydrocephalus secondary to SAH. Subgroup analysis looking at individual aetiologies or drainage methods was not possible due to the small sample size. CSF dynamics and hydrocephalus from acute insults to previously healthy brains are likely to differ from chronic pathology-limiting comparisons. The choice of drain device (tunnelled-EVD, Bolt-EVD, or LD) was consultant-specific and non-standardised, reflecting real-life practice in the absence of clear guidelines. Likewise, the method for the gradual weaning cohort was surgeon-specific and not uniform. Although attempts were made to attempt to account for this variability through multivariate techniques, they would be better analysed through a highly controlled, randomised trial. Indeed, the heterogeneity across this cohort highlights why more evidence is required to guide practice and that this study, at least in an exploratory format, may be used to guide a more focused, randomised trial.

The lack of randomisation between early and late-challenged patients may have led to a selection bias, with ‘well’ patients being judged to be ready for an early challenge and, hence, a shorter stay. Furthermore, this study did not assess differences between continuous and intermittent drainage, as the centre uniformly uses a continuous drainage approach. Finally, an assessment of overall patient outcome and disability was beyond the scope of the study, although it may have been heavily influenced and overshadowed by pathology rather than drainage parameters.

## 5. Conclusions

This study adds to the limited body of evidence on weaning CSF diversion. In support of the ASA and Neurocritical Care Society, we recommend initiating a drain challenge early, via a rapid wean, to reduce the length of stay, mechanical complications, and possibly infections. The consequences of temporary CSF diversion have significant implications in terms of patient and financial outcomes, but the quality of evidence regarding weaning remains poor. Further randomised multicentre trials and national databases of practice are required to allow definitive conclusions to be drawn.

## Figures and Tables

**Table 1:** Summary of patient demographics, device type, and wean characteristics. (* Stroke refers to ischaemic stroke or hemorrhagic stroke either in the parenchyma or ventricle).

**Patients**	69
**Total number of drains inserted**	80
**Mean age in years (SD)**	59.4 (46.7–72.1)
**Gender (F/M [%])**	36/33 [52.1/47.8]
**Primary pathology (%)**	Aneurysmal SAH = 41 (59.4) * Stroke = 15 (21.7) Tumour = 9 (13.0) Non-aneurysmal SAH = 2 (2.9) Meningitis = 2 (2.9)
**Drain type (%)**	Bolt EVD = 48 (69.5) Tunnel EVD = 13 (18.8) Lumbar drain = 8 (11.6)
**Drainage system (%)**	Becker = 46 (66.7) LiquoGuard = 19 (27.5) Mixed = 4 (5.8)
**Mean consecutive length of drainage (median, range)**	13.3 days (13, 2–25)
**Wean method (%)**	Rapid = 39 (56.5) Gradual = 19 (27.5) Removed = 11 (15.9)
**Mean duration before first drain clamp (median, range)**	9.9 days (8, 2–22)
**Number shunted (median time to shunt, range)**	32 (16.5, 2–53 days)

**Table 2:** Summary of key results. (KW = Kruskal–Wallis, LOS = hospital length of stay, MWU = Mann–Whitney U, Rank = Spearman Rank coefficient).

			Univariate		Multivariate (Adjusted)		
	Predictor	Outcome	Stat	*p*	Stat	Coef (CI)	*p*
**LOS**	Time to first clamp	Hospital LOS (days)	Rank = 0.34	<0.01	t = 2.73	1.25 (0.33–2.16)	<0.01
		Drain duration (days)	Rank = 0.63	<0.0001	-		-
**Shunt**	Wean method	Time to shunt (days)	KW = 7.96	0.02	t = −1.93 (removed)	−12.40 (−22.87–−0.73)	0.06
	Time to first clamp	Time to shunt (days)	Rank = 0.36	0.04	t = 2.46	1.03 (0.17–1.88)	0.02
**CNS infection**	Wean method	Time to first infection (days)	KW = 11.1	<0.01	t = −5.0 (rapid wean)	−6.31 (−9.07–−3.54)	<0.0001
**Mechanical complications**	Wean method	Number of discrete episodes	MWU = 42	0.04	t = −2.19 (rapid wean)	−0.54 (−1.04–−0.05)	0.03

## Data Availability

Data available on request.

## Supplementary Materials

The following supporting information can be downloaded at: https://www.mdpi.com/article/10.3390/neurosci5040030/s1.

## References

[1] Dey M., Stadnik A., Riad F., Zhang L., McBee N., Kase C., Carhuapoma J.R., Ram M., Lane K., Ostapkovich N. (2015). Bleeding and Infection with External Ventricular Drainage: A Systematic Review in Comparison with Adjudicated Adverse Events in the Ongoing Clot Lysis Evaluating Accelerated Resolution of Intraventricular Hemorrhage Phase III (CLEAR-III IHV) Trial. Neurosurgery.

[2] Hughes J.D., Puffer R., Rabinstein A.A. (2015). Risk factors for hydrocephalus requiring external ventricular drainage in patients with intraventricular hemorrhage. J. Neurosurg..

[3] Lyke K.E., Obasanjo O.O., Williams M.A., O’Brien M., Chotani R., Perl T.M. (2001). Ventriculitis Complicating Use of Intraventricular Catheters in Adult Neurosurgical Patients. Clin. Infect. Dis..

[4] Roach J., Gaastra B., Bulters D., Shtaya A. (2019). Safety, Accuracy, and Cost Effectiveness of Bedside Bolt External Ventricular Drains (EVDs) in Comparison with Tunneled EVDs Inserted in Theaters. World Neurosurg..

[5] Chung D.Y., Leslie-Mazwi T.M., Patel A.B., Rordorf G.A. (2017). Management of External Ventricular Drains After Subarachnoid Hemorrhage: A Multi-Institutional Survey. Neurocrit. Care.

[6] Capion T., Lilja-Cyron A., Bartek J., Forsse A., Logallo N., Juhler M., Mathiesen T. (2020). Discontinuation of External Ventricular Drainage in Patients with Hydrocephalus Following Aneurysmal Subarachnoid Hemorrhage—A Scandinavian Multi-institutional Survey. Acta Neurochir..

[7] Connolly E.S., Rabinstein A.A., Carhuapoma J.R., Derdeyn C.P., Dion J., Higashida R.T., Hoh B.L., Kirkness C.J., Naidech A.M., Ogilvy C.S. (2012). Guidelines for the Management of Aneurysmal Subarachnoid Hemorrhage. Stroke.

[8] Fried H.I., Nathan B.R., Rowe A.S., Zabramski J.M., Andaluz N., Bhimraj A., Guanci M.M., Seder D.B., Singh J.M. (2016). The Insertion and Management of External Ventricular Drains: An Evidence-Based Consensus Statement. Neurocrit. Care.

[9] Klopfenstein J.D., Kim L.J., Feiz-Erfan I., Hott J.S., Goslar P., Zabramski J.M., Spetzler R.F. (2004). Comparison of rapid and gradual weaning from external ventricular drainage in patients with aneurysmal subarachnoid hemorrhage: A prospective randomized trial. J. Neurosurg..

[10] Dey M., Jaffe J., Stadnik A., Awad I.A. (2012). External Ventricular Drainage for Intraventricular Hemorrhage. Curr. Neurol. Neurosci..

[11] Chau C.Y.C., Craven C.L., Rubiano A.M., Adams H., Tülü S., Czosnyka M., Servadei F., Ercole A., Hutchinson P.J., Kolias A.G. (2019). The Evolution of the Role of External Ventricular Drainage in Traumatic Brain Injury. J. Clin. Med..

[12] Valadka A.B. (2015). Are External Ventricular Drains Better than Parenchymal Intracranial Pressure Monitors in Trauma Patients?. World Neurosurg..

[13] Gu J., Wu H., Chen X., Feng J., Gao G., Jiang J., Mao Q. (2020). Intracranial Pressure during External Ventricular Drainage Weaning Is an Outcome Predictor of Traumatic Brain Injury. Biomed. Res. Int..

[14] Von Elm E., Altman D.G., Egger M., Pocock S.J., Gøtzsche P.C., Vandenbroucke J.P., Initiative S. (2007). The Strengthening the Reporting of Observational Studies in Epidemiology (STROBE) statement: Guidelines for reporting observational studies. Lancet.

[15] Sedgwick P., Greenwood N. (2015). Understanding the Hawthorne effect. BMJ Br. Med. J..

[16] Chung D.Y., Thompson B.B., Kumar M.A., Mahta A., Rao S.S., Lai J.H., Tadevosyan A., Kessler K., Locascio J.J., Patel A.B. (2021). Association of External Ventricular Drain Wean Strategy with Shunt Placement and Length of Stay in Subarachnoid Hemorrhage: A Prospective Multicenter Study. Neurocrit. Care.

[17] Jabbarli R., Pierscianek D., RÖlz R., Reinhard M., Oppong M.D., Scheiwe C., Dammann P., Kaier K., Wrede K.H., Shah M. (2018). Gradual External Ventricular Drainage Weaning Reduces The Risk of Shunt Dependency After Aneurysmal Subarachnoid Hemorrhage: A Pooled Analysis. Oper. Neurosurg..

[18] Rao S.S., Chung D.Y., Wolcott Z., Sheriff F., Khawaja A.M., Lee H., Guanci M.M., Leslie-Mazwi T.M., Kimberly W.T., Patel A.B. (2020). Intermittent CSF drainage and rapid EVD weaning approach after subarachnoid hemorrhage: Association with fewer VP shunts and shorter length of stay. J. Neurosurg..

[19] Pandit A.S., Palasz J., Nachev P., Toma A.K. (2022). Mechanical Complications of External Ventricular and Lumbar Drains. World Neurosurg..

[20] Parry S.M., Puthucheary Z.A. (2015). The impact of extended bed rest on the musculoskeletal system in the critical care environment. Extrem. Physiol. Med..

[21] Karic T., Røe C., Nordenmark T.H., Becker F., Sorteberg W., Sorteberg A. (2017). Effect of early mobilization and rehabilitation on complications in aneurysmal subarachnoid hemorrhage. J. Neurosurg..

[22] Perry A., Graffeo C.S., Kleinstern G., Carlstrom L.P., Link M.J., Rabinstein A.A. (2020). Quantitative Modeling of External Ventricular Drain Output to Predict Shunt Dependency in Aneurysmal Subarachnoid Hemorrhage: Cohort Study. Neurocrit. Care.

[23] Park P., Garton H.J.L., Kocan M.J., Thompson B.G. (2004). Risk of Infection with Prolonged Ventricular Catheterization. Neurosurgery.

[24] Camacho E.F., Boszczowski Í., Basso M., Jeng B.C.P., Freire M.P., Guimarães T., Teixeira M.J., Costa S.F. (2011). Infection rate and risk factors associated with infections related to external ventricular drain. Infection.

[25] Jamjoom A.A.B., Joannides A.J., Poon M.T.-C., Chari A., Zaben M., Abdulla M.A., Roach J., Glancz L.J., Solth A., Duddy J. (2018). Prospective, multicentre study of external ventricular drainage-related infections in the UK and Ireland. J. Neurol. Neurosurg. Psychiatry.

[26] Dakson A., Kameda-Smith M., Staudt M.D., Lavergne P., Makarenko S., Eagles M.E., Ghayur H., Guo R.C., Althagafi A., Chainey J. (2022). A nationwide prospective multicenter study of external ventricular drainage: Accuracy, safety, and related complications. J. Neurosurg..

