# Supplementary material for: Discontinuation of Cerebro-Spinal Fluid (CSF) Drainage in Acute Hydrocephalus: A Prospective Cohort Study and Exploratory Data Analysis
Source: NeuroSci. 2024 Oct 8;5(4):396–406. doi: 10.3390/neurosci5040030 (PMC11503252; doi:10.3390/neurosci5040030)
Supplement: Supplementary file 1 [file neurosci-05-00030-s001.zip › neurosci-3140480-supplementary.pdf]

Supplementary Material

Supplementary Results

Influence of wean method and speed on hospital length of stay

Supplementary Table S1. Differences in hospital length of stay by wean method

Wean method	Median hospital length of stay in days (IQR)	Statistic
Gradual	30 (24 - 41.5)	KW, statistic = 0.14, p = 0.93
Removed	27 (23.5 - 44.5)	
Rapid	28 (19 - 47)	

Variables suspected of influencing hospital length of stay included: age, sex, wean method, time to clamp, diagnosis, whether the patient had a CNS infection and suffered an episode of radiological vasopasm. These were used to identify the best subset (Supplementary Table S2).

Supplementary Table S2. Best subset multiple linear regression model based on Akaike (AIC) and Bayesian Information Criterion (BIC) showing adjusted effect of time to first clamp on length of stay. (CI = confidence interval)

Variable	Coefficient (CI)	p
Intercept	12.27 (1.16 - 23.38)	0.03
Days to first clamp	1.25 (0.33 - 2.16)	0.008
Any mechanical failure	6.04 (-2.55 - 14.63)	0.17
Stroke diagnosis	16.63 (4.97 - 28.29)	0.006
Any vasospasm	6.70 (-3.10 - 16.49)	0.18
Model parameters: Adj. R^2 = 0.195, AIC = 564, BIC = 575		

Influence of weaning on shunt frequency and timing

Supplementary Table S3. Association between wean method, shunt frequency and timing

Wean method	Shunted	Non-shunted	Statistic	Median time to shunt in days (IQR)	Statistic
Gradual	6	12	X^2, statistic =	23.5 (19.3 - 25.5)	KW, statistic = 7.96, p =

Removed	5	6	2.09, p = 0.35	10.0 (8.0 - 11.0)	0.02<br><br>Dunn:<br>Gradual vs. removed,, p = 0.01<br><br>Rapid vs. removed, p = 0.08<br>Gradual vs. rapid, p = 0.13
Rapid	21	18		17.0 (12.0 - 24.0)	

Variables suspected of influencing time to shunt included: age, sex, wean method, time to clamp, diagnosis, drainage collecting system and drain type. These were used to identify the best subset (S. Table S4).

Supplementary Table S4. Best subset multiple linear regression model showing factors associated with time to shunt based on Akaike and Bayesian Information Criterion. (CI = confidence interval)

**Variable**	**Coefficient (CI)**	**p**
Intercept	-12.65 (-37.5 – 12.22)	0.213
Age	0.33 (0.02 – 0.64)	0.03
Days to clamp	1.03 (0.17 – 1.88)	0.02
Sex	7.74 (-1.38 –16.9)	0.09
Wean method: removal	-12.40 (-22.87 – -0.73)	0.06
Model parameters: Adj. R^2 = 0.23, AIC = 247, BIC = 254		

Supplementary Figure S1. Kaplan-Meier cumulative incidence curves showing probability of shunt treatment by different wean methods for the first month of admission

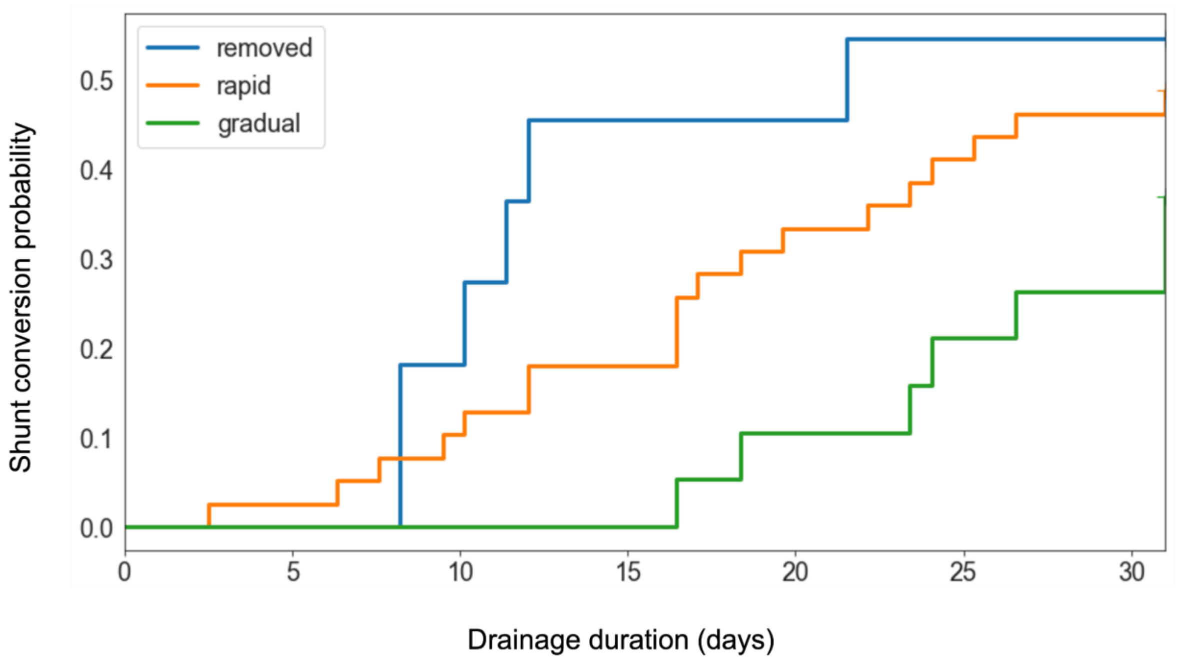

Influence of weaning on risk of CNS infection

Supplementary Table S5. Association between wean method and occurrence of CNS infection. FE = Fisher-Exact with Freeman-Hamilton extension. Only significant or trending post-hoc p-values are reported.

Wean method	CNS infection	No CNS infection	Statistic	Median time to CNS infection in days (IQR)	Statistic
Gradual	5	14	FE, p = 0.23	11 (10 - 13)	KW, statistic = 11.15, p = 0.003<br><br>Dunn:<br>Gradual vs. rapid,, p = 0.008<br><br>Gradual vs. removed, p = 0.03
Removed	1	10		2 (2 - 2)	
Rapid	14	25		5.5 (4 - 7)	

Variables suspected of influencing occurrence of CNS infection included: age, sex, wean method, time to clamp, diagnosis, and presence of another infection. These were used to identify the best subset (S. Table S6).

Supplementary Table S6. Best subset multiple linear regression model showing factors associated with time to CNS infection based on Akaike and Bayesian Information Criterion. (CI = confidence interval)

Variable	Coefficient (CI)	p
Intercept	6.00 (-0.16 - 12.17)	0.06
Stroke diagnosis	2.94 (0.16 - 5.72)	0.04
Meningitis diagnosis	-1.86 (-6.76 - 3.04)	0.43
Tumour diagnosis	0.73 (-2.54 - 4.00)	0.64
Wean method: rapid	-6.31 (-9.07 - -3.54)	<0.001
Wean method: removal	-12.56 (-18.02 - -7.12)	<0.001
Days to clamp	0.02 (-0.19 - 0.23)	0.87
Age	0.09 (-0.01 - 0.19)	0.09
Model parameters: Adj. R^2 = 0.70, AIC = 93, BIC = 100		

Influence of weaning on mechanical complications

Supplementary Table S7. Association between wean method and occurrence of mechanical complications. Only significant or trending post-hoc p-values are reported. *Excluded from analysis (KW = Kruskal Wallis, MWU = Mann-Whitney U)

Wean method	Mechanical complication	No mechanical complication	Statistic	Median episodes	Statistic	Median time to first mechanical failure (IQR)	Statistic
Gradual	9	10	X^2, statistic = 1.84, p = 0.17	0 (0 - 2)	MWU, statistic = 427, p = 0.04	1 (0 - 4.3)	MWU, statistic = 37.5, p = 0.57
*Removed	7	4		2 (0 - 2.5)		1 (0.3 - 3.8)	
Rapid	10	29		0 (0 - 0.5)		0 (0 - 4)	

Variables suspected of influencing occurrence of mechanical complication included: age, sex, wean method, time to clamp, diagnosis, and presence of another infection. These were used to identify the best subset (Supplementary Table S8).

Supplementary Table S8. Best subset multiple linear regression model showing factors associated with episodes of mechanical failure on Akaike and Bayesian Information Criterion. (CI = confidence interval)

Variable	Coefficient	p
Intercept	0.56 (0.11 – 1.00)	0.02
Collecting system: LiquoGuard	0.33 (-0.18 – 0.83)	0.20
Collecting system: mixed	1.88 (0.99 – 2.77)	<0.001
Drain type: lumbar drain	0.16 (-0.55 – 0.87)	0.66
Drain type: tunnelled EVD	1.04 (0.49 – 1.59)	<0.001
Wean method: rapid	-0.54 (-1.04 – -0.05)	0.03
Wean method: removed	0.30 (-0.36 – 0.95)	0.37
Model parameters: Adj. R^2 = 0.40, AIC = 180, BIC = 196		
